# Clinical trials of probiotics: Current outlook

**DOI:** 10.1002/fsn3.3884

**Published:** 2023-12-07

**Authors:** Haitham Al‐Madhagi, Abir Alramo

**Affiliations:** ^1^ Biochemical Technology Program Dhamar University Dhamar Yemen; ^2^ Division of Microbiology, Department of Plant Biology Aleppo University Aleppo Syria

**Keywords:** clinical trials, FDA, microbiota, probiotics

## Abstract

The current investigation provides a summary of the available clinical trials using probiotics as therapeutic worldwide and their fate.

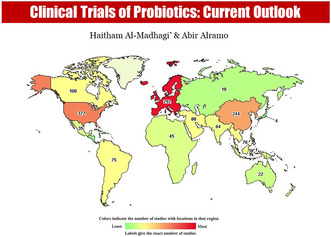

## 1 LETTER

Many microorganisms inhabit different systems of the human body and live in a harmonic, mutualistic relationship. The most important niche in which the microbiome inhabits is the gut. The gut microbiota involves various strains of non‐pathogenic bacteria, archaea, and yeasts creating a microbial community that interplays different roles in host physiology such as metabolism, immunity, and even nervous system homeostasis. Accordingly, scientists have noticed such integral part of the gut microbiota for human benefit and redirect their potential use as biotherapy (called probiotics) (Dahiya & Nigam, 2023). A plenty of studies proved the efficacy of probiotics for the treatment or management of a variety of illnesses. The ingested probiotics exert their actions in different ways: (i) compete with the pathogenic bacteria for inhabitance, (ii) compete for nutrients, and (iii) secrete antibiotics that eradicate the pathogenic bacteria. Additionally, probiotics can aid in the digestion of fibers that host enzymes cannot deal with. Furthermore, they also release exopolysaccharides, peptides, and metabolites with potent pharmaceutical activity. Moreover, some reports confirmed the reshaping role of the probiotics toward the host immune system (Balthazar et al., 2022). Most of the probiotics tested are derived from animal raw milk and fermented foods. These include cattle raw milk, buffalo raw milk, yogurt, and different kinds of pickles. It should be noted that the ingested bacteria to be used as a probiotics must withstand the highly acidic medium of stomach as well as the bile acids and digestive enzymes cocktail in the intestine (Abid, Farid, et al., 2022; Abid, Ghazanfar, et al., 2022; Hameed et al., 2022; Naeem et al., 2018).

New drug discovery and development, including biopharmaceuticals such as probiotics, is a multifaceted and lengthy process that requires several stages over more than a decade (7–10 years on average) and entails an expenditure of approximately $1–2 billion per drug to achieve the Food and Drug Administration (FDA) approval (Hinkson et al., 2020). The candidate compound has to undergo four consecutive phases of clinical trials. Phase I assesses the safety profile of the compound in a small cohort of subjects (<50). Phase II evaluates the efficacy of the compound in a larger sample size (<100). Phase III conducts a large‐scale comparison (hundreds of participants) with the standard of care. Finally, if the compound passes all these phases successfully, it receives the approval and its long‐term effects are monitored (thousands of participants). However, the duration from drug approval to market launch is usually about 1.5 years on average (Dowden & Munro, 2019). Progressing to phase I is considered a remarkable achievement for pharmaceutical companies and academic institutions, as it implies surpassing stringent stages of drug optimization and preclinical testing. Nevertheless, approximately 90% of drug approval failures happen during phase I progression (Takebe et al., 2018). The global vision of the availability of clinical trials testing probiotics is not satisfied. Hence, we sought to give an outline and analyze the ongoing clinical trials that deal with probiotics throughout the world.

**FIGURE 1:**
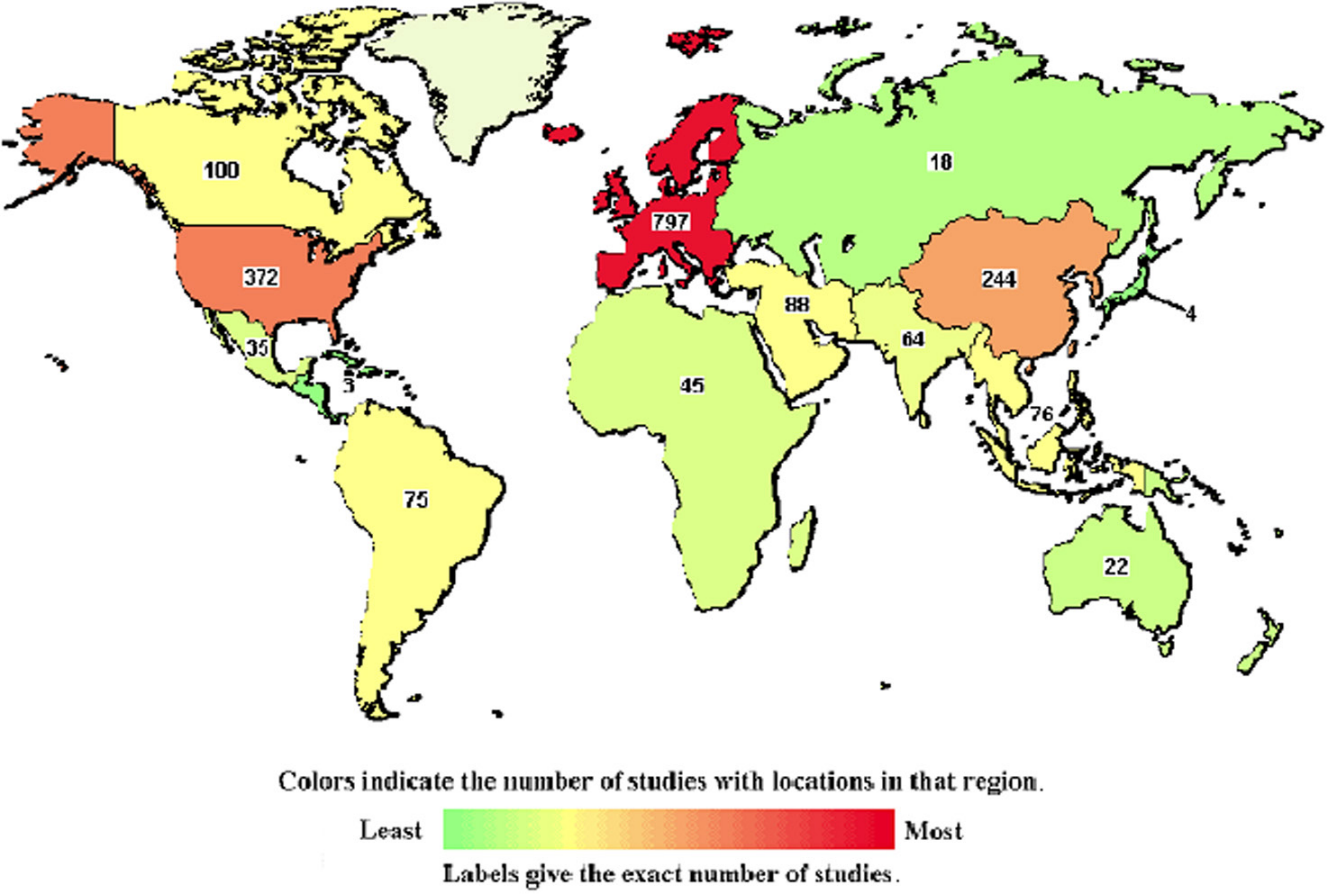
Worldwide distribution of the registered clinical trials concerning probiotics.

Probiotics available trials (https://clinicaltrials.gov/) are distributed throughout the world with Europe as the major contributor (797), followed by North America (503, from which 372 from USA alone) and East Asia (244) (Figure 1). Moreover, Middle East is on increasing rate (88). Currently, there are 2147 registered clinical trials worldwide that use prebiotics as a novel medical intervention to manage/treat a broad range of diseases. These include multiple types of infections (viral including COVID‐19, bacterial, fungal, and parasitic), antibiotic resistance, gastrointestinal disease (the most frequent are irritable bowel syndrome, various types of diarrhea, and constipation), malignancies, neural/behavioral disorders (such as Parkinson's disease, bipolar disease, anorexia, and depression), diabetes and obesity, liver diseases, congenital disease (like lactose intolerance), aging, preterm infants, inflammatory infestations, and as dietary supplements.

**FIGURE 2:**
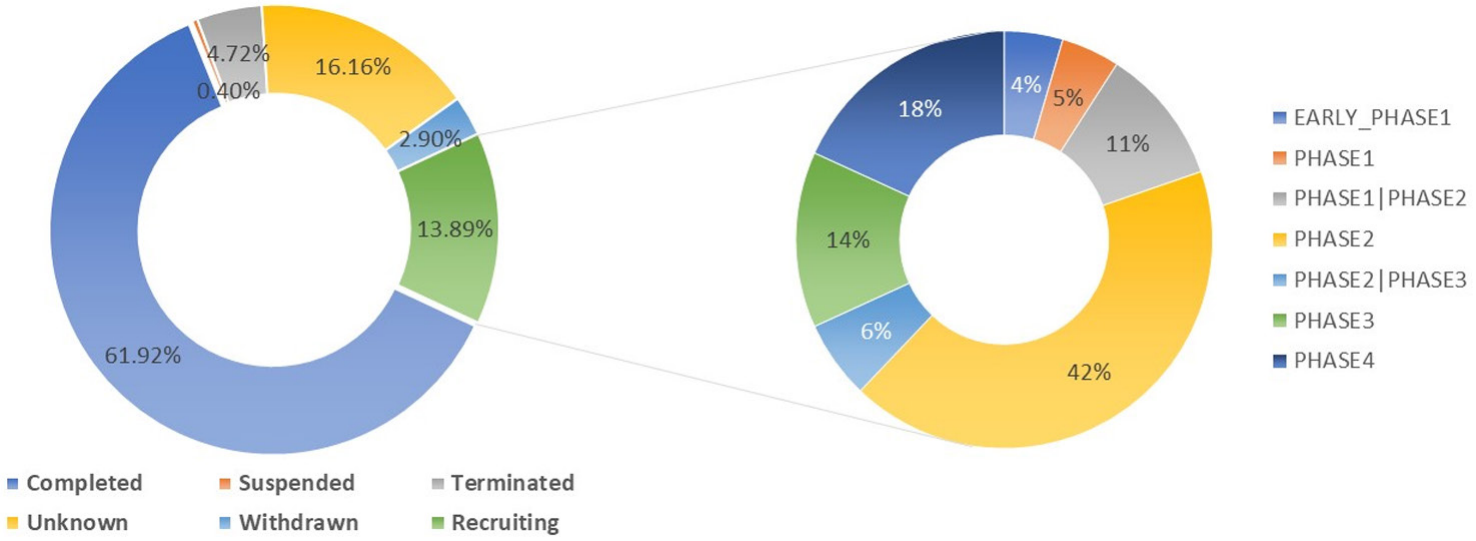
Fate of the available probiotics clinical trials and the corresponding phases of the recruiting ones.

According to the analyzed data, 61% of clinical trials were successfully completed in all phases. Only 13% are recruiting, while the remaining percentage is distributed to suspended (0.4%), terminated (4.72%), and withdrawn (2.90%). This failure is usually traced to several factors, including poor clinical efficacy (40%–50%), unbearable toxicity (30%), pharmacokinetics issues (10%–15%), and poor financial and management strategies (10%) (Sun et al., 2022). Notably, 16.16% of all clinical trials have “unknown” fate. Such huge percentage creates a big question mark about the surveillance of the clinical trials and their output. Among all of the ongoing clinical trials about probiotics, only 18% succeeded in phase IV while the majority are in the phase II (42.42%) (Figure 2).

Despite the establishment of 372 trials in the USA alone, no FDA approval for any of the tested probiotics was certified. Nonetheless, the USA market is full of healthcare products, certain cosmetics, and dietary supplements (but not as drugs) due to no need for FDA approval prior to marketing of these products (Lee et al., 2019). On the contrary, Japan has approved the safety and potency of some probiotics in spite of the scarce number of clinical trials owing to the variation in drug legislations among the countries (Hickey, 2017).

The examined probiotics involved numerous bacterial species, but the main ones were *Lactobacilli* and *Bifidobacteria*. *Lactobacillus* species included *L. rhamnosus*, *L. plantarum*, *L. helveticus*, *L. casei*, and *L. paracasei*, while *B. longum*, *B. infantis*, and *B. brevel* were the main used *Bifidobacteria* strains. Indeed, these bacterial species can resist harsh conditions of the gut, besides its modulatory properties of the immune system (Vera‐Santander et al., 2023). However, *Bacillus coagulans*, *B. clausii*, and *Streptococcus salivarius* were used in other trials. Some trials used a cocktail of two or three types of above‐mentioned strains.

## AUTHOR CONTRIBUTIONS

**Haitham Al‐Madhagi:** Conceptualization (lead); data curation (equal); writing – original draft (lead). **Abir Alramo:** Data curation (equal); writing – review and editing (lead).

## FUNDING INFORMATION

This paper received no funding.

## CONFLICT OF INTEREST STATEMENT

None declared.

## Data Availability

All data were extracted from https://clinicaltrials.gov/.
